# Autophagic Cell Death Is Induced by Acetone and Ethyl Acetate Extracts from *Eupatorium odoratum In Vitro*: Effects on MCF-7 and Vero Cell Lines

**DOI:** 10.1100/2012/439479

**Published:** 2012-04-30

**Authors:** Faizah Bt. Harun, Syed Mohsin Syed Sahil Jamalullail, Khoo Boon Yin, Zulkhairi Othman, Anita Tilwari, Prabha Balaram

**Affiliations:** ^1^Institute for Research in Molecular Medicine (INFORMM), Universiti Sains Malaysia, Health Campus, 16150 Kubang Kerian, Kelantan, Malaysia; ^2^School of Health Sciences, Universiti Sains Malaysia, Health Campus, 16150 Kubang Kerian, Kelantan, Malaysia

## Abstract

*Eupatorium odoratum (EO)* contains many biologically active compounds, the anticancer effects of which are not well documented. This study evaluates the cytotoxic effects and mechanism of action of *EO* extracts on MCF-7 and Vero cell lines. Evaluation of the cytotoxic activity using MTT assay, morphological alterations, and apoptosis were carried out. Autophagy was evaluated by LC3-A protein expression. Cytotoxic activity, membrane blebbing and ballooning at 24 hours, replacement by mass vacuolation, and double membrane vesicles mimicking autophagy and cell death were observed in the cancer cells. No apoptosis was observed by DNA fragmentation assay. Overexpression of LC3-A protein indicated autophagic cell death. Cell cycle analysis showed G0 and G2/M arrest. The Vero cells did not show significant cell death at concentrations <100 **μ**g/mL. These results thus suggest that acetone and ethyl acetate extracts of *EO* induce cell death through induction of autophagy and hold potential for development as potential anticancer drugs.

## 1. Introduction

The urge to find new compounds among plants to fight cancer has become a matter of great interest among researchers. Plants harbor many chemical compounds with known or unknown potential activities against diseases.

*Eupatorium odoratum, *also known as *Chromolaena odorata *(L.) King and Robinson, is a wildly growing free standing shrub from the family of Asteracea. Growing in a wet habitat, this plant has three-nerved leaves opposing each other, shaped into deltoid to oval lanceolate margin, and has long pointed tips. The white-colored flower of this plant is tubular. Previous reports observed it to posses various biological activities including antimicrobial and anticancer effects. Local name for this plant include “pokok kapal terbang,” rumput putih (Indonesia), Siam weed, and others. Traditional use of the leaves from this plant to reduce aches and pains has been practiced by villagers [1]. People in Machang, Kelantan, and Malaysia use this plant to treat wounds, uterus-related problems, and to stop bleeding [2]. Hence it would be interesting to study the anticancer effects of its extracts which would help in evaluating the active principles as anticancer drugs.

 Breast cancer is one of the most debilitating disease of modern times [3]. The main modes of therapy used for control of this disease are surgery and chemotherapy with varying degrees of failure due to the high resistance to chemotherapy [4]. The treatment responses are especially dismal for hormone-independent cancers [4]. Hence, research into novel therapeutic compounds for these cancers is warranted. 

Plant products induce cell death by a variety of mechanisms. The most common mechanisms reported are programmed cell death (PCD)-type I (apoptosis), PCD type II autophagic cell death, and necrosis [5]. PCD type I is a mode of self cannibalism which involves individual cells [6] does not cause inflammation to the neighboring cells, and has become a mechanism of interest in drug research. However, for certain cells in which the mechanism of cell death is caspase independent like in MCF-7 cells, other mechanisms which might differ from PCD type I could be in place such as PCD type II autophagy. Autophagic cell death is divided into three types: macroautophagy, microautophagy, and chaperone-mediated autophagy. Main features of this PCD are that it involves formation of double membrane vesicles and also autolysosomes, and can be visualized under phase contrast microscope as double membrane vacuole like structures inside the cytoplasm surrounding the organelles. These vesicles have been shown to fuse with lysosomes (containing lysozyme) to form autolysosomes and the organelles inside are processed and converted into ATP to be further used for cell metabolism [7, 8]. In certain treatments with plant extracts inducing excessive autophagic mechanism, a situation arises when no organelles are left inside the cells to be used as energy. This leads to cell death, and DNA laddering might also be seen at later phases during the digestion of the nucleus by the endonuclease.

A number of drug compounds such as Tamoxifen, Rapamycin, sodium butyrate, and SAHA induce autophagy in cancer cells. Compounds, isolated from plants such as resveratrol, oleandrin, and triterpenoid saponins have also been shown to exhibit autophagy in a variety of cancer cells [9–11]. The mode of action of the extracted compounds depends on the parts of the plants used and the mode of extraction. Using Acetone as the solvent, compounds belonging to lipid soluble or phenolic compound classes are extracted, while ethyl acetate extracts more of phenols and flavonoids which are heat stable compounds. Many of the compounds extracted by these solvents are reported to have potent anticancer activity. Luteolin and phenolic compounds present in plants of this species have shown evidence of moderate toxicity against NCI-H187 cells and weak toxicity against human breast cancer cells [12, 13]. However, little is known about its effects on different tumor cell lines, especially caspase-3 deficient cell lines. In addition, the mechanisms of cell death induced by this plant are not well described or reported.

In this study, the elucidation of cytotoxicity effects on epithelial carcinoma cell lines and the mechanism of death using evaluation of morphological alterations, DNA-laddering assay and LC3-A protein expression, showed the acetone and ethyl acetate extracts of *Eupatorium odoratum*, to induce cell death predominantly through autophagy. 

## 2. Materials and Methods

### 2.1. Cell Culture Preparation

Caspase-3-deficient human metastatic mammary carcinoma cells (MCF-7) and Vero cells were maintained in RPMI-1640 (Hyclone, USA) with glutamine and supplemented with 10% fetal bovine serum (FBS) (Hyclone, USA) and Penicillin-Streptomycin (Gibco, Invitrogen) at 37°C in 5% CO_2_ humidified atmosphere incubator (Thermo Forma, USA).

### 2.2. Plant Material

The plant* Eupatorium odoratum *was collected by means of universal sampling. The leaves were separated from the fresh plant and used for preparation of the extracts.

### 2.3. Preparation of *E. odoratum* Extracts

Extracts were prepared using Soxhlet extraction method. Briefly, leaves of *E. odoratum* were rinsed with tap water, washed with 5% ethanol, followed by rinsing with distilled water. Leaves were then dried at 37°C in dry oven and ground into powder and extracted using five different solvents following the nonpolar to polar solvent regime, namely, petroleum ether, ethyl acetate, acetone, methanol, and water. Solvents were diminished from the extracts by reduced-pressure evaporation using rotary evaporator (Heidolph, Germany). Aqueous extract was dried using freeze drying method and kept at −20°C.

Excluding aqueous extract, other plant extracts were dissolved in DMSO to obtain final concentration of 50 mg/mL, filter sterilized with 0.20 *μ*M PVDF membrane and stored at −20°C.

Working solution was prepared by diluting the stock solution with RPMI-1640 containing 10% FBS into nine different concentrations (5 *μ*g/mL, 10 *μ*g/mL, 20 *μ*g/mL, 25 *μ*g/mL, 50 *μ*g/mL, 100 *μ*g/mL, 250 *μ*g/mL, 500 *μ*g/mL, and 1000 *μ*g/mL) just before use.

### 2.4. Cytotoxicity Assay

Cytotoxicity of plant extracts on MCF-7 cell line was tested using MTT (3-(4,5-dimethylthiazol-2-yl)-2,5-diphenyltetrazolium bromide, a tetrazole) cytotoxicity assay. Briefly, cells (1.25 × 10^4^ cells/mL) were seeded into sterile 96-well flat bottom culture plates (Nunc, Germany). After 24 hours of incubation, plant extracts were added into respective culture wells (final concentration ranging from 2.5 to 500 *μ*g/mL) and cultured for three time intervals: 24, 48, and 72 hours. Tamoxifen at the same concentrations as the plant extract including the IC_50_ value was added as the control drug to assess cell death. 4 hours before the end of the culture period, media from each well was removed, and cell surface was washed with phosphate-buffered saline (PBS) pH 7.4 two times. 15 *μ*L MTT reagent was added, and the plate was incubated at 37°C for 4 hours. Formazan crystals were then solubilized with 100 *μ*L DMSO, and the plate was incubated in dark for 2 hours. Finally, absorbance was measured at 595 nm wavelength using ELISA reader (Thermo Scientific, USA). Vero cells grown and treated in the same manner were used as the control for the MTT assays. IC_50_ was calculated using the Graph Pad Prism software. All assays were carried out in triplicate, and the mean values were recorded.

### 2.5. Morphological Study

MCF-7 cells at 1.25 × 10^5^ cells/mL were cultured in 96 well cell culture plates and incubated in 5% CO_2_ incubator at 37°C for 24 hours to obtain cells in the log phase of its growth. Subsequently, 100 *μ*L plant extracts at the IC_50_ concentrations was added into the culture wells in triplicate. No extract was added in the wells adjacent to the ones in which the extract was added. These wells served as controls for each period of incubation. Cells were incubated in incubator for respective culture periods (24, 48, or 72 hours). After each treatment, culture media from the well was removed and transferred into separate tubes. Cells remaining inside the wells were observed under a phase contrast microscope and the picture was captured. For fluorescent microscopic analysis, 25 *μ*L of cell suspension was mixed with 25 *μ*L of acridine orange/ethidium bromide working solution (1x) and left for 2 minutes at room temperature to allow absorption of dye into cells. Cells were then observed under white light of fluorescent microscope, and finally under blue light (450 nm). Assessment was carried out for features such as membrane blebbing, nuclear condensation, and other features of apoptosis and cytoplasmic and nuclear changes. Pictures observed were captured and edited using Q-Capture software.

### 2.6. DNA Extraction

Control and treated cells seeded in triplicate inside 96-well cell culture plates were harvested using trypsinization method. Cell suspensions were then centrifuged at 2000 rpm for 5 minutes to pellet the cells. Supernatant was removed, and the pellet was resuspended in 1 mL cell lysis buffer; 10% SDS, 2 *μ*L RNase (100 *μ*g/mL), and incubated at 56°C for 2 hours with brief vortexing for 10 seconds each for 15 minutes. 10 *μ*L of Proteinase K (20 mg/mL) was added to the tubes and incubated at 37°C for 2 hours with brief vortexing for 10 seconds each for 15 minutes followed by two volumes of cold isopropanol added into the mixture to precipitate the DNA. The tubes were left for 10 minutes at room temperature. Tubes containing DNA was then centrifuged at 13000 rpm for 20 minutes in refrigerated centrifuge at 4°C. DNA pellet formed was washed with 70% ethanol and centrifuged. Supernatant was totally removed and DNA pellet was air dried at room temperature and finally resuspended in 20–50 *μ*L tris-EDTA (TE) buffer. The prepared DNA was checked for purity and concentration using a nanodrop. A ratio of 1.6 and above was considered pure enough to carry out the experiments. DNA was stored at −20°C until use.

### 2.7. DNA-Laddering Assay

The DNA isolated from cells treated with the plant extracts at different time intervals, and untreated cells were electrophoresed on 1.5% agarose gel at 100 mV for 1 hour 30 minutes. 100 bp DNA marker was run in the first lane to act as a guide for assessing ladder formation.

### 2.8. Cell Cycle Analysis

Cells after treatment with the extracts were trypsinized and collected using method similar to subculturing of the cells. Cell number was adjusted to 2 × 10^6^ cells/mL suspension. The cells were then transferred into 5 mL polystyrene round bottom tubes and spun at 1000 rpm for 5 minutes. Supernatant was discarded, and pellet was resuspended in cold PBS, pH 7.2. Cells were fixed by adding 4 mL cold absolute ethanol. 100 *μ*L of 200 *μ*g/mL RNase was added, and the tube was incubated at 37°C for about 30 minutes to remove RNase. 100 *μ*L of 1 mg/mL Propidium iodide (PI) (Sigma Aldrich, USA) was added, and tubes were incubated in the dark at room temperature (25°C) for 5 to 10 minutes. The cells were analysed using FACS-SCAN (BD, Bio Sciences) within 3 hours of PI addition. Data was analyzed using FACSDiva software Version 6.1.2.

### 2.9. Immunocytochemistry

Cells after treatment were harvested via trypsinization. Cell smears were made by spreading the cells evenly on the microscopic slides. The smears were dried at room temperature for 10 minutes and immersed into cold acetone (−20°C) for 20 minutes to fix the cells. Slides with cell smears were kept at −80°C until use. The slides were brought to room temperature before start of immunostaining. In order to block endogenous peroxidase activity, which might interfere with the staining, slides were incubated in 3% H_2_O_2_ in TBS for 10 minutes and then rinsed with TBS-T20 2-3 times. 200 *μ*L of 1 : 100 diluted LC3-A antibody (Abcam, USA) was added, and the slides were incubated for 1 hour followed by rinsing with TBS-20 and incubated with biotinylated universal anti-immunoglobulin in phosphate-buffered saline (PBS) containing stabilizing protein with 0.015 mol/L sodium azide (Dako, USA). After 30 minutes of incubation, slides were washed with TBS-20 to remove nonspecific binding of the antibody. 2 drops of streptavidin conjugated with horseradish peroxidase (HRP) in PBS (Dako, USA) were added onto the slides and incubated for another 30 minutes. Positive cells showing LC3-A protein expression were detected by developing the slides using 3,3′-diaminobenzidine (DAB) chromogen containing hydrogen peroxide. Slides were left for 10 minutes to allow color development and then counterstained with hematoxylin and rinsed with running tap water to remove excess dye. Slides were subjected to dehydration process by immersing in ascending grades of absolute ethanol and finally in xylene for two minutes and mounted using Cytosealer. Expression of LC3A was evaluated under a light microscope and graded positive/negative in comparison to the respective control slides.

## 3. Results

### 3.1. Cytotoxicity Assay

**Figure 1:**
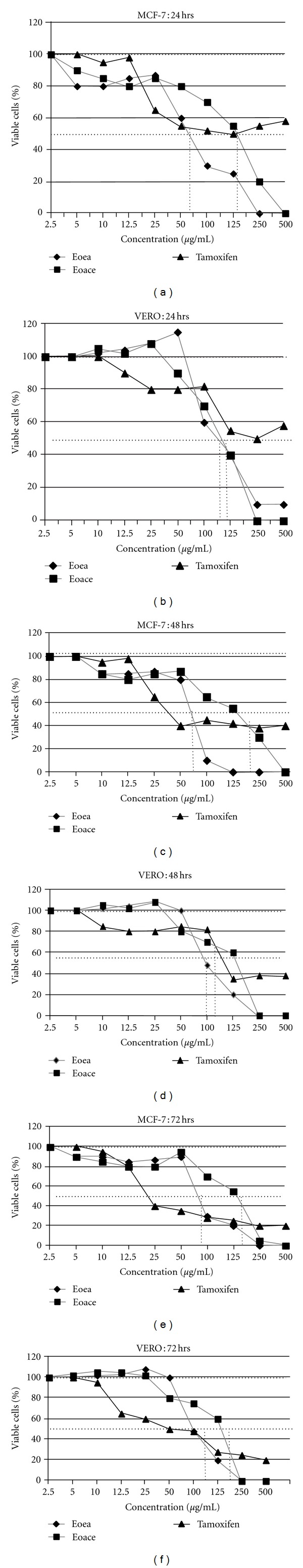
Graph showing cell death induced by acetone and ethyl acetate extracts of *E. odoratum *on MCF-7 and Vero cell culture *in vitro *at various time periods: 24 hours, 48 hours, and 72 hours.

 MTT cytotoxicity assay was done to assess the cytotoxic potential of EOea and EOace on MCF-7 cancer cells and Vero cells. On treatment with EOea, the MCF-7 cells showed an increased rate of cell death at a lower concentration of the extracts when compared to that in the Vero cells (Figure 1 and Table 1). For MCF-7 cells, the IC_50_ recorded for ethyl acetate extracts of EO were 65.72 *μ*g/mL, 83.88 *μ*g/mL and 92.84 *μ*g/mL and that for the acetone extracts were 133.9 *μ*g/mL at 24 hours, but increased to 163.0 *μ*g/mL at 48 hours and 147.8 *μ*g/mL at 72 hours (Figure 1 and Table 1). The IC_50_ values for both the extracts were lower than that for tamoxifen. Cell death could be observed in Vero cells mainly at concentrations >100 *μ*g/mL.

### 3.2. Morphological Observations

**Figure 2:**
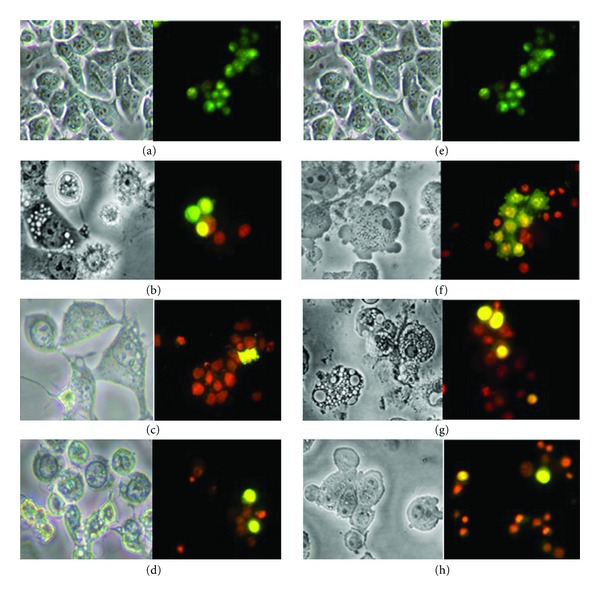
Phase contrast and fluorescent microscopic analysis of MCF-7 cells treated with EOea and EOace at different time periods. (a) and (e) untreated MCF-7 cells, (b), (c), (d) MCF-7 cells treated with EOea at 24, 48, and 72 hours, (f), (g), (h) cells treated with EOace at 24, 48, and 72 hours.

Morphological alterations in the treated cells were observed in both EOea- and EOace-treated MCF-7 cells.The observations revealed the effects of the EOace to be more prominent in treated MCF-7 cells when compared to untreated cells (Figure 2). At 24 hours of treatment, enlargement of the cells which was more prominent in EOace (Figures 2(b) and 2(f)) was observed. 50–60% of the cells showed membrane blebbing (indicated by small protrusions of the membrane) at this period. At 48 hours, blebbing and ballooning of the membrane were prominent in almost all the cells. Cells showed extensive vacuolation in the cell cytoplasm (Figures 2(c) and 2(g)), indicating autophagy like mechanism of cell death. Autophagosome like structures were clearly seen in the cells at 24–48 hours of treatment in both EOea- and EOace-treated cells (Figures 2(b), 2(f), 2(c), and 2(g)). Similar observations were also seen in the acridine orange-ethidium bromide staining where the blebbing could be seen distinctly at 24–48 hours of incubation with the extract, and shrinkage and cell death were observed at 72 hours (Figures 2(d) and 2(h)). The cells were rounded, shrunk, and showed signs of detachment from the surface of the wells and cell death. No presence of apoptotic bodies could be observed at any time point. No apoptotic changes or vacuolation were observed in the Vero cells at the IC_50_ concentration.

### 3.3. DNA Laddering and Immunocytochemistry Assays

**Figure 3:**
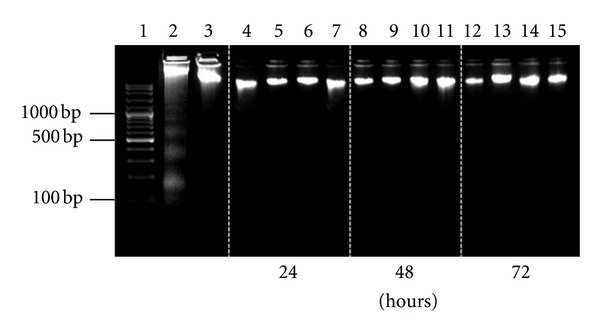
DNA laddering assay showing no DNA fragmentation or necrosis in MCF-7 cells treated with EOace and EOea at various time intervals., Lanes 1: DNA 100 bp ladder; 2: positive control; 3: negative control (untreated cells); 4 and 5: EOea-treated MCF-7 cells—24 hours; 6 and 7: EOace-treated MCF-7 cells—24 hours; 8 and 9: EOea-treated MCF-7 cells—48 hours; 10 and 11: EOace-treated MCF-7 cells—48 hours; 12 and 13: EOea-treated MCF-7 cells—72 hours; 14 and 15: EOace-treated MCF-7 cells—72 hours.

DNA laddering assay showed the genomic DNA to be localized near the wells and did not show any laddering (Hallmark of apoptosis) or smearing indicative of DNA shearing and necrosis (Figure 3) when compared to the positive control.

### 3.4. Cell Cycle Analysis

**Figure 4:**
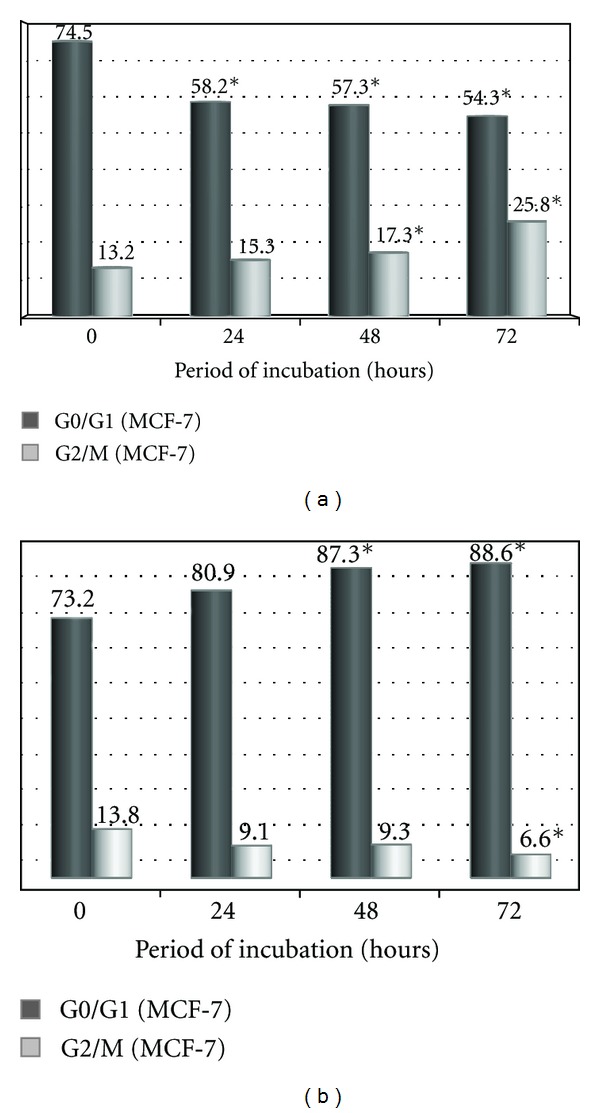
Histogram showing the proportion of cells in G0/G1 and G2/M phases of the cell cycle at different periods of treatment with EOea and EOace. (a)MCF-7 cells treated with EOea; (b) MCF-7 cells treated with EOace.

To test if EOea and EOace cause a stage-dependant mechanism of inhibition, asynchronised Vero cells and MCF-7 cells were analysed by flow cytometry of stained DNA following treatment with the extracts. The proportion of cell population in the three phases of the cell cycle are shown in Figures 4(a) and 4(b). A significant increase was observed in the G2/M phase accompanied by a reduction in the G0/G1 phase in the MCF-7 cells on treatment with EOea (Figure 4(a)). Treatment with EOace showed an increase in the G0/G1 phase accompanied by a decrease in the G2/M phase (Figure 4(b)). No significant difference was observed in the Vero cells treated for the same period. The effect of EOea on G0/G1 was significant (*P* < 0.05) as early as 24 hours of treatment while the EOace was slower affecting the cell cycle significantly (*P* < 0.05) only around 48 hours of treatment.

### 3.5. Expression of LC3-A Protein

The absence of apoptotic changes, positive cytotoxic effect, and morphological appearance resembling autophagy prompted us to evaluate the expression, using immunohistochemical analysis, of LC3-A protein which is involved during the process of autophagy. Human brain tissue was used as the positive control for expression of LC3-A as directed in the manufacturer's brochure.

**Figure 5:**
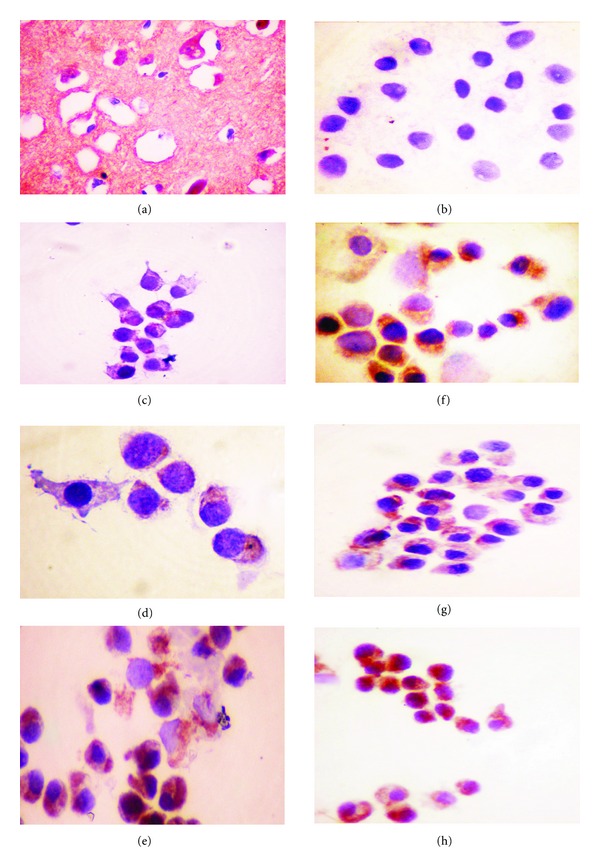
Immunocytochemical staining of MCF-7 cells treated with EOace and EOea at various time periods. Positive control: (a) Human brain tissue, (b)Untreated MCF-7 cells, (c) EOace treated MCF-7 cells at 24 hours, (d) 48 hours and (e) 72 hours. (f) EOea treated MCF-7 cells at 24 hours, (g) 48 hours and (h) 72 hours. Positive expression of LC3-A is indicated by the reddish brown staining.

 Treatment of MCF-7 cells with EOea and EOace showed positive expression of LC3-A protein, which was localized in the cytoplasm of the cells. As seen in the morphological evaluation, the staining was more prominent in the EOace-treated cells when compared to the EOea-treated cells. No expression was observed in the untreated MCF-7 cells, and the cells had clear cytoplasm (Figure 5(b)). At 24 hours, many cells showed the expression of LC3-A, but with mild staining of the cytoplasm. In the case of EOea-treated cells, the number of cells showing positive staining for LC3-A was much less in number when compared to the EOace-treated cells (Figures 5(c) and 5(f)). After 48 hours of treatment, almost all the cells showed positive staining of the cells in the EOace-treated cells while 75% of the cells were positive in the EOea-treated cells (Figures 5(d) and 5(g)). At 72 hours, the expression was much higher with both extracts and displayed intense staining for the protein (Figures 5(e) and 5(h)). 

## 4. Discussion

Plants used in folk and traditional medicines have been accepted as leads for therapeutic drug development in modern medicine.* Eupatorium odoratum* was chosen for this study due to its use as an anticancer and wound-healing agent among the natives of Malaysia and in other parts of the world [1, 2]. No documentation of its mechanism of action was found in literature, and hence this study evaluated the cytotoxic potential, the mechanism of cell death, and effects on cell cycle of acetone and ethyl acetate extracts of this plant on MCF-7 cells *in vitro*. Studies have observed the presence of a large number of bioactive compounds in the acetone and ethyl acetate extracts of plants including flavanoids, phenolic compounds, triterpenoids, flavonoids, [14–16] which can be extracted out by these solvents. These compounds are present in a number of food items and hold great potential as drug candidates due to their safety, low toxicity, and wide acceptance.

Previous studies have isolated flavonoids, chalcones, flavones, essential oils, and other biological compounds from different parts of the plants of this family [12, 17–19], even though not *Eupatorium odoratum* per se. It was also observed that different compounds differed in their target of action in bringing about cell death [20, 21]. Antibacterial activity, antioxidant activity, and anticancer activity have been documented in the extracts of these plants [12, 13] with no details of the mechanisms involved. Eupallinin A, a naturally occurring phytoalexin from Eupatorium Chinense L., was found to inhibit growth of Leukemia HL60 cells *in vitro* [17]. 

Caspase-3-deficient breast cancer cell line MCF-7 was used as the test system in this study which was prompted by the requirement of more effective treatment for the increasing incidence of breast cancers worldwide. The results of the present study showed potent cytotoxic effects on MCF-7 cells with ethyl acetate and acetone extracts of this plant. The ethyl acetate extract was active at a lower concentration (minimum IC_50_—65.72 *μ*g/mL) when compared to the acetone extract at a minimum IC_50_ of 133.9 *μ*g/mL as is evident from Table 1, and Figure 1. The IC_50_ value was found to be higher than that specified by NCI, USA for categorization of a pure compound as anticancer agent but lower than that of the standard drug tamoxifen. This could be due to the fact that crude extracts were used in this study due to the exploratory nature of the study, and inappropriate combinations of components in the extract would result in nullifying effects towards each other [16]. The reduction in viable cell number was evident as early as 24 hours of treatment with both the extracts. The morphological effects were more prominent in the acetone extract-treated cells showing extensive blebbing and vacuolation (Figures 2(a)–2(h)) suggesting autophagic mechanism of cell death. Strong expression of LC3-A, a protein involved in autophagic mechanism, was observed in immunohistochemical analysis of the cells after treatment (Figures 5(c)–5(h)). No signs of apoptotic cell death could be observed in morphological evaluation and DNA fragmentation assays confirming the absence of apoptosis in these cells. MCF-7 cells lack functional caspase-3 due to a 47 bp deletion in exon 3 of the gene which could probably lead to the inability of these cells to undergo caspase-dependant apoptosis [22, 23]. 

Apoptosis, even though has been the most studied cell death mechanism in cancer cells, is not the sole response to DNA damage. Autophagic cell death has recently been described as an alternate mechanism of cell death which is a degradative pathway characterized by formation of membrane-bound vesicles in the cytoplasm, known as autophagic vacuoles and gradual death of the cell on persistence of the stress agent. This has been implicated as a defense mechanism to protect against infectious agents, drug resistance, survival during starvation, cytotoxic stimuli, and stressful conditions. Continued accumulation of the autophagic vacuoles due to persistence or high levels of the stress agent results in negation of the protective effect and leads to autophagocytosis and eventual cell death. Autophagy may also be followed by apoptosis in certain situations, especially when the cells have an active caspase-3 [24]. A number of plant compounds such as camptothecin [25], oridonin [26] has been shown to induce autophagy in these cells.

Antitumor effects may be brought about by altered biochemical mechanisms, influencing cell proliferation, induction of cell cycle arrest at various cell cycle checkpoints, enhanced apoptosis, and altered expression of key enzymes. The target of action varies with different plant-derived compounds determined by factors such as their structure and position of sugar moieties. In the present study, the influence of treatment of MCF-7 cells on cell cycle progression was assessed by flow cytometry. The results show that the two extracts targeted different stages of the cell cycle suggesting different targets of action. EOea induced a G2/M phase arrest accompanied by a reduction in the G0/G1 phase of the cell cycle while the acetone extract induced a G0/G1 arrest with a concomitant decrease in the G2/M phase. The relationship between cell cycle and autophagy has not been reported in great detail. An accumulation of cells in G1 phase has been reported while undergoing autophagy following withdrawal of growth factors in cell culture [27]. Reports show that stabilisation of p27 inhibits cyclin-dependant kinase activity and defects in Cdk2- and Cdk4-increased autophagy [28] suggesting a link between autophagy and G1 arrest [29]. Induction of autophagy has been observed to reduce cellular levels of p53,  a major function of which is to act as a repressor of autophagy [30] leading to an accumulation of cells in the G1 phase. The observation of a G0/G1 arrest on treatment with EOace suggests the Antitumor effect of this extract to be through regulation of proteins such as p53 or the cyclin-dependant kinases.

The observation of an increase in the G2/M phase of the cell cycle on treatment with the ethyl acetate extract is similar to that observed by Kuo et al. on treating breast cancer cells with plumbagin [31]. Their experiments have shown that the inhibition of AKT pathways plays a role in inducing G2 arrest in MDA-MB-231 cells by bringing about accumulation of inactive phospho-Cdc2 and phospho-Cdc25C, leading to subsequent G2 arrest. Significant inhibition of cell cycle progression was observed by 6 hours of treatment with a clear increase in the percentage of cells in the G2/M phase when compared to that in normal controls. These findings thus suggest that the reduction observed in the viable cells following treatment with EOea is due to autophagic cell death and is associated with cell cycle arrest in the G2/M phase.

In conclusion, the present observations provide preliminary data to show that acetone and ethyl acetate extracts of *Eupatorium odoratum* have potent cytotoxic activity against MCF-7 cells. The data further emphasize that the mechanism underlying cell death by these extracts is due to autophagy and cell cycle arrest. The data also suggest that the target of action of the active compound in the two extracts is different. This calls for further studies on the active components for proper assessment of their chemotherapeutic properties and possible development as promising anticancer drugs.

## Figures and Tables

**Table 1:** Cytotoxic concentrations (IC_50_ values) of EOea and EOace extracts against MCF-7 and Vero cells at different periods of treatment.

	IC50 value (*μ*g/mL)								
Cells	Time (hours)								
	24			48			72		
	EOea	EOace	TAM	EOea	EOace	TAM	EOea	EOace	TAM
Vero cells	115.74	121.96	>500	105.04	115.49	91.78	114.49	125.94	102.42
MCF-7 cells	65.72	133.9	138.7	83.88	163.03	85.77	92.84	147.8	45.88

## References

[1] Ong HC, Norzalina J (1999). Malay herbal medicine in Gemencheh, Negri Sembilan, Malaysia. *Fitoterapia*.

[2] Ong HC, Nordiana M (1999). Malay ethno-medico botany in Machang, Kelantan, Malaysia. *Fitoterapia*.

[3] Hisham AN, Yip CH (2004). Overview of breast cancer in Malaysian women: a problem with late diagnosis. *Asian Journal of Surgery*.

[4] Bange J, Zwick E, Ullrich A (2001). Molecular targets for breast cancer therapy and prevention. *Nature Medicine*.

[5] Díaz LF, Chiong M, Quest AFG, Lavandero S, Stutzin A (2005). Mechanisms of cell death: molecular insights and therapeutic perspectives. *Cell Death and Differentiation*.

[6] Bold RJ, Termuhlen PM, McConkey DJ (1997). Apoptosis, cancer and cancer therapy. *Surgical Oncology*.

[7] Hung CC, Davison EJ, Robinson PA, Ardley HC (2006). The aggravating role of the ubiquitin-proteasome system in neurodegenerative disease. *Biochemical Society Transactions*.

[8] Levine B, Kroemer G (2008). Autophagy in the pathogenesis of disease. *Cell*.

[9] Kondo Y, Kanzawa T, Sawaya R, Kondo S (2005). The role of autophagy in cancer development and response to therapy. *Nature Reviews Cancer*.

[10] Anthony WO, Tan L, Boitano AE, Sorenson DR, Aurora A, Liu JR (2004). Resveratrol-induced autophagocytosis in ovarian cancer cells. *Cancer Research*.

[11] Meschini S, Condello M, Marra M, Formisano G, Federici E, Arancia G (2007). Autophagy-mediated chemosensitizing effect of the plant alkaloid voacamine on multidrug resistant cells. *Toxicology in Vitro*.

[12] Suksamrarn A, Chotipong A, Suavansri T (2004). Antimycobacterial activity and cytotoxicity of flavonoids from the flowers of *Chromolaena odorata*. *Archives of Pharmacal Research*.

[13] Srinivasa Rao K, Chaudhury PK, Pradhan A (2010). Evaluation of anti-oxidant activities and total phenolic content of *Chromolaena odorata*. *Food and Chemical Toxicology*.

[14] Hassan SM, Ghareib HR (2009). Bioactivity of *Ulva lactuca* L. acetone extract on germination and growth of lettuce and tomato plants. *African Journal of Biotechnology*.

[15] Gilani AH, Atta-ur-Rahman (2005). Trends in ethnopharmacology. *Journal of Ethnopharmacology*.

[16] Dai J, Mumper RJ (2010). Plant phenolics: extraction, analysis and their antioxidant and anticancer properties. *Molecules*.

[17] Yuan J, Yang J, Miao J (2005). Chemical constituents of *Eupatorium odoratum*. *Chinese Traditional and Herbal Drugs*.

[18] Bose PK, Chakrabarti P, Chakravarti S, Dutta SP, Barua AK (1973). Flavonoid constituents of *Eupatorium odoratum*. *Phytochemistry*.

[19] Owolabi MS, Ogundajo A, Yusuf KO (2010). Chemical composition and bioactivity of the essential oil of *Chromolaena odorata* from Nigeria. *Records of Natural Products*.

[20] Salucci M, Stivala LA, Maiani G, Bugianesi R, Vannini V (2002). Flavonoids uptake and their effect on cell cycle of human colon adenocarcinoma cells (Caco2). *British Journal of Cancer*.

[21] Ishiguro K, Ando T, Maeda O (2007). Ginger ingredients reduce viability of gastric cancer cells via distinct mechanisms. *Biochemical and Biophysical Research Communications*.

[22] Yang XH, Sladek TL, Liu X, Butler BR, Froelich CJ, Thor AD (2001). Reconstitution of caspase 3 sensitizes MCF-7 breast cancer cells to doxorubicin- and etoposide-induced apoptosis. *Cancer Research*.

[23] Fazi B, Bursch W, Fimia GM (2008). Fenretinide induces autophagic cell death in caspase-defective breast cancer cells. *Autophagy*.

[24] Rodriguez-Rocha H, Garcia-Garcia A, Panayiotidis MI, Franco R (2011). DNA damage and autophagy. *Mutation Research*.

[25] Abedin MJ, Wang D, McDonnell MA, Lehmann U, Kelekar A (2007). Autophagy delays apoptotic death in breast cancer cells following DNA damage. *Cell Death and Differentiation*.

[26] Cui Q, Tashiro S-I, Onodera S, Minami M, Ikejima T (2007). Autophagy preceded apoptosis in oridonin-treated human breast cancer MCF-7 cells. *Biological and Pharmaceutical Bulletin*.

[27] Lum JJ, Bauer DE, Kong M (2005). Growth factor regulation of autophagy and cell survival in the absence of apoptosis. *Cell*.

[28] Liang J, Shao SH, Xu ZX (2007). The energy sensing LKB1-AMPK pathway regulates p27kip1 phosphorylation mediating the decision to enter autophagy or apoptosis. *Nature Cell Biology*.

[29] Tasdemir E, Maiuri MC, Tajeddine N (2007). Cell cycle-dependent induction of autophagy, mitophagy and reticulophagy. *Cell Cycle*.

[30] Tasdemir E, Maiuri MC, Galluzzi L (2008). Regulation of autophagy by cytoplasmic p53. *Nature Cell Biology*.

[31] Kuo PL, Hsu YL, Cho CY (2006). Plumbagin induces G2-M arrest and autophagy by inhibiting the AKT/mammalian target of rapamycin pathway in breast cancer cells. *Molecular Cancer Therapeutics*.

